# UGT79B31 is responsible for the final modification step of pollen-specific flavonoid biosynthesis in *Petunia hybrida*

**DOI:** 10.1007/s00425-017-2822-5

**Published:** 2017-12-06

**Authors:** Eva Knoch, Satoko Sugawara, Tetsuya Mori, Ryo Nakabayashi, Kazuki Saito, Keiko Yonekura-Sakakibara

**Affiliations:** 1 RIKEN Center for Sustainable Resource Science, 1-7-22, Suehiro-cho, Tsurumi-ku, Yokohama, 230-0045 Japan; 2 Graduate School of Pharmaceutical Sciences, Chiba University, 1-8-1, Inohana, Chuo-ku, Chiba, 260-8675 Japan

**Keywords:** Arabidopsis, Glycosyltransferase, Flavonol, Male sterility, Petunia

## Abstract

***UGT79B31*** **encodes flavonol 3-** ***O*** **-glycoside: 2″-** ***O*** **-glucosyltransferase, an enzyme responsible for the terminal modification of pollen-specific flavonols in** ***Petunia hybrida***.

Flavonoids are known to be involved in pollen fertility in petunia (*P*. *hybrida*) and maize (*Zea mays*). As a first step toward elucidating the role of flavonoids in pollen, we have identified a glycosyltransferase that is responsible for the terminal modification of petunia pollen-specific flavonoids. An in silico search of the petunia transcriptome database revealed four candidate UDP-glycosyltransferase (UGT) genes. *UGT79B31* was selected for further analyses based on a correlation between the accumulation pattern of flavonol glycosides in various tissues and organs and the expression profiles of the candidate genes. Arabidopsis *ugt79b6* mutants that lacked kaempferol/quercetin 3-*O*-glucosyl(1 → 2)glucosides, were complemented by transformation with *UGT79B31* cDNA under the control of Arabidopsis *UGT79B6* promoter, showing that UGT79B31 functions as a flavonol 3-*O*-glucoside: 2″-*O*-glucosyltransferase in planta. Recombinant UGT79B31 protein can convert kaempferol 3-*O*-galactoside/glucoside to kaempferol 3-*O*-glucosyl(1 → 2)galactoside/glucoside. UGT79B31 prefers flavonol 3-*O*-galactosides to the 3-*O*-glucosides and rarely accepted the 3-*O*-diglycosides as sugar acceptors. UDP-glucose was the preferred sugar donor for UGT79B31. These results indicated that *UGT79B31* encodes a flavonoid 3-*O*-glycoside: 2″-*O*-glucosyltransferase. Transient expression of UGT79B31 fused to green fluorescent protein (GFP) in *Nicotiana benthamiana* showed that UGT79B31 protein was localized in the cytosol.

**Electronic supplementary material:**

The online version of this article (10.1007/s00425-017-2822-5) contains supplementary material, which is available to authorized users.

## Introduction

Flavonoids are major plant secondary metabolites with over 9000 compounds distributed widely throughout the plant kingdom (Markham 1988; Richardson 1989; Williams and Grayer 2004; Anderson and Markham 2006). The biosynthetic pathways leading to the core skeletons have been well studied in terms of natural product chemistry, genetics and molecular biology, whereas pathways for subsequent modification steps such as glycosylation, acylation and methylation are being elucidated in several plant species (Anderson and Markham 2006; Saito et al. 2013). Flavonoids play important roles as pigments, UV protectants, attractants for pollinators, phytoalexins, signaling molecules and regulators of fertility and auxin transport (Falcone Ferreyra et al. 2012; Xu et al. 2015). Nevertheless, the enormous chemical diversity of flavonoid structures and the intricate distribution patterns in plant tissues and species make it difficult to correlate specific flavonoid structures, including modification patterns, with their physiological functions.

As one of a few exceptions, a relationship between pollen-specific flavonol glycosides and pollen fertility is well established (Mo et al. 1992; van der Meer et al. 1992; Ylstra et al. 1992). Pollen of flavonoid-deficient mutants of petunia (*P*. *hybrida*) are unable to germinate, resulting in male sterility (Mo et al. 1992; Napoli et al. 1999). Likewise, maize (*Z*. *mays*) mutants deficient in flavonoids are also male sterile (Pollak et al. 1995). This phenotype was rescued by the exogenous addition of flavonol aglycones such as kaempferol and quercetin, indicating that flavonoids are essential for functional pollen in petunia and maize (Mo et al. 1992). In the pollen of petunia mutants, exogenously added flavonol aglycones are rapidly converted into the flavonol diglucosides, kaempferol/quercetin 3-*O*-glucosyl(1 → 2)galactosides that are identical to those accumulating in the wild type (Zerback et al. 1989; Vogt and Taylor 1995). Diglycosides of quercetin and isorhamnetin including quercetin 3-*O*-rhamnosyl(1 → 2)glucoside are prominent flavonoids in maize pollen (Ceska and Styles 1984).

Flavonol 3-*O*-diglycosides with a 1 → 2 inter-glycosidic linkage often accumulate as major flavonoids in pollen. Kaempferol and/or quercetin 3-*O*-glucosyl(1 → 2)glucosides (3-*O*-sophorosides) are the dominant flavonol glycosides in pollen from *Arabidopsis thaliana* (Stracke et al. 2010; Yonekura-Sakakibara et al. 2014) and plants in the Juglandaceae, Betulaceae, Fagaceae and Oleaceae families (Pratviel-Sosa and Perchero 1972). Interestingly, the Arabidopsis *tt4* and *ugt79b6* mutants were fertile, even though they lack chalcone synthase, the first committed enzyme in flavonoid biosynthesis, and pollen-specific UDP-glycosyltransferase (UGT), the enzyme responsible for the terminal modification of pollen flavonoids, respectively (Yonekura-Sakakibara et al. 2014). In Arabidopsis pollens, flavonols are converted to flavonol 3-*O*-glucosides by UGT78D2, a flavonoid 3-*O*-glucosyltransferase, and are subsequently modified to flavonol *3*-*O*-glucosyl(1 → 2)glucosides by UGT79B6, a flavonol 3-*O*-glucoside: 2″-*O*-glucosyltransferase. *UGT78D2* is expressed throughout the plant but *UGT79B6* expression is specific to the tapetum and microspores of developing anthers (Yonekura-Sakakibara et al. 2014). In petunia, flavonols are converted to flavonol 3-*O*-galactosides by a pollen-specific flavonol 3-*O*-galactosyltransferase (F3GalTase, F3GalT) (Miller et al. 1999), and further modified to flavonol *3*-*O*-glucosyl(1 → 2)galactosides by an unknown flavonol 3-*O*-galactoside: 2″-*O*-glucosyltransferase (F3GT) (Vogt and Taylor 1995). It was suggested that F3GalT and/or F3GT may be associated with a pollen membrane (Vogt and Taylor 1995).

To elucidate the role of flavonoids in pollen fertility, we have identified a gene encoding a glycosyltransferase, F3GT, that is responsible for the final modification step in the biosynthesis of petunia pollen-specific flavonoids. Based on an in silico search of the petunia transcriptome database, flavonol analyses in various organs and expression profiles of the candidate genes, we focused on UGT79B31. In vitro characterization of UGT79B31 and functional complementation of Arabidopsis mutants that lacked flavonoid diglycosides indicated that *UGT79B31* encodes flavonoid 3-*O*-glycoside: 2″-*O*-glucosyltransferase.

## Materials and methods

### Plant materials

Seeds of *P. hybrida* inbred line, V26 (kindly provided by Dr. M. Nakayama, NARO Institute of Floricultural Science, Tsukuba, Ibaraki, Japan) were used. Petunia seeds were sown on one-half-strength MS-agar medium containing 2% (w/v) sucrose and placed in a 25 °C growth chamber with a light intensity of 70 µmol of photons m^2^ s^−1^ and a 16 h light/8 h dark photoperiod. After 8 weeks, the seedlings were transferred to sterile vermiculite and acclimated. After acclimation, plants were transferred to soil and grown for 3 months in a greenhouse.

*Arabidopsis thaliana* accession Columbia-0 (Col-0; Lehle Seeds, Texas, USA) was used as the wild type. The Arabidopsis TILLING line CS95581 (*ugt79b6*-*3*) was previously described (Yonekura-Sakakibara et al. 2014). *Nicotiana benthamiana* seeds were grown in soil at 22 °C with a 16 h light/8 h dark photoperiod.

### Database search

A BLAST search with the tblastn program was conducted using *UGT79B6* from *A. thaliana* as a query for the *P. hybrid*a databases in the categories/database (Transcriptome projects/*P. hybrida* var. Mitchell transcriptome Villarino 2014 Contigs, SGN Unigenes current version/*P*. *hybrida* Unigenes and SGN ESTs/*P*. *hybrida* SGN mRNA sequences) at the Sol Genomics Network (https://solgenomics.net/). Sequences with the following scores were used for further analyses: Categories/Database, Transcriptome projects/*P. hybrida* var. Mitchell transcriptome Villarino 2014 Contigs, > 200; SGN Unigenes current version/*P*. *hybrida* Unigenes > 80; SGN ESTs/*P*. *hybrida* SGN mRNA sequences > 80.

For a comprehensive search for homologues of UGT79B31, UGT92A1 and SGN210759, a BLAST search in the Sol Genomics Network (https://solgenomics.net/) was conducted as follows: Categories, *Petunia* sps. Genome (current version); Database, *Petunia axillaris* v1.6.2 proteins/*Petunia inflata* v1.0.1 proteins; Program, blastp. Sequences showing over 40% identity with the query were used for the analyses.

### Flavonoid profiling by UPLC/PDA/QTOF/MS

Fresh samples were extracted with 5 μl of 80% MeOH containing 2.5 µM lidocaine and 10-camphor sulfonic acid per mg fresh weight using a mixer mill with zirconium beads for 7 min at 18 Hz and 4 °C. After centrifugation for 10 min, the supernatant was filtered using an HLB μElution plate (Waters). The extracts (1 μl) were analyzed using LC-QTOF-MS (LC, Waters Acquity UPLC system; MS, Waters Xevo G2 Q-Tof). Analytical conditions were as follows: LC column, Acquity bridged ethyl hybrid (BEH) C18 (1.7 μm, 2.1 mm × 100 mm, waters); solvent system, solvent A [water including 0.1% (v/v) formic acid] and solvent B [acetonitrile including 0.1% (v/v) formic acid]; gradient program, 90% A/10% B at 0 min, 90% A/10% B at 0.1 min, 80% A/20% B at 25 min, 0% A/100% B at 25.1 min, 0% A/100% B at 27.5 min, 90% A/10% B at 27.6 min and 90% A/10% B at 30.0 min; flow rate, 0.3 ml/min; column temperature, 40 °C; photodiode array, 200–500 nm; flavonoid detection, 320 nm; MS detection: capillary voltage, + 3.0 keV, cone voltage, 25.0 V, source temperature, 120 °C, desolvation temperature, 450 °C, cone gas flow, 50 l/h; desolvation gas flow, 800 l/h; collision energy, 6 V; mass range, *m*/*z* 50‒2000; scan duration, 1.0 s; inter-scan delay, 0.014 s; data acquisition, centroid mode; polarity, positive; Lockspray (leucine enkephalin): scan duration, 1.0 s; inter-scan delay, 0.1 s. MS/MS data were acquired in the ramp mode in the following analytical conditions: (1) MS: mass range, *m*/*z* 50–1500; scan duration, 1.0 s; inter-scan delay, 0.014 s; data acquisition, centroid mode; and (2) MS/MS: mass range, *m*/*z* 50–1500; scan duration, 0.1 s; inter-scan delay, 0.014 s; data acquisition, centroid mode; collision energy, ramped from 10 to 50 V. In this mode, MS/MS spectra of the top 10 ions (> 10,000 counts) in an MS scan were automatically obtained. If the ion intensity was less than 10,000, MS/MS data acquisition was not performed and moved to the next top 10 ions.

### Degenerate PCR

**Fig. 1:**
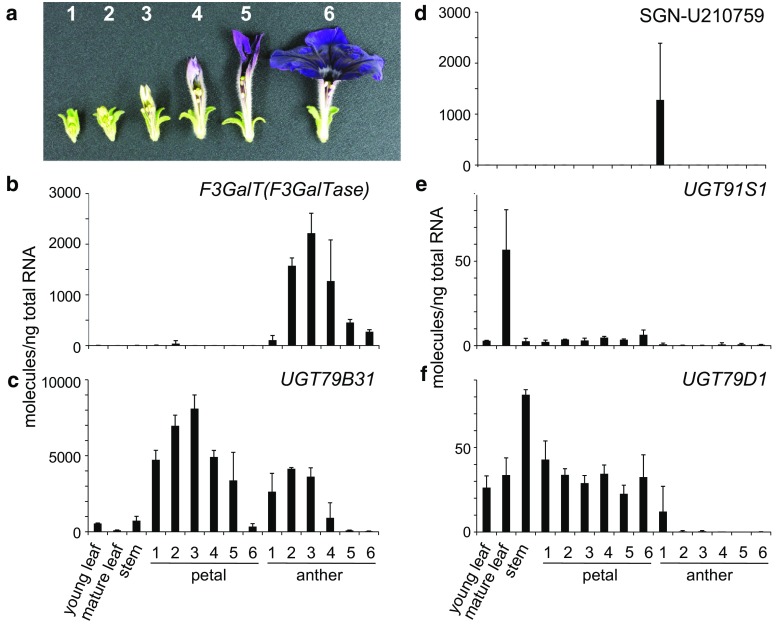
Expression analysis of candidate UGT genes in petunia organs. **a** Petunia flower/pollen developmental stages: stage 1, 0–15 mm buds; stage 2, 16–25 mm buds; stage 3, 26–35 mm buds; stage 4, 36–50 mm buds; stage 5, 51–60 mm buds; stage 6, opened/opening flowers with indehiscent pollens. Flower developmental stages 1–5 in this study correspond to stages 1–2, 3–4, 5–6, 7–8, 9–10, respectively, as described previously (Vogt and Taylor 1995). **b**–**f** Expression profiles of petunia *F3GalT*(*F3GalTase*) (**b**), *UGT79B31* (**c**), SGN-U210759 (**d**), *UGT91S1* (**e**) and *UGT79D1* (**f**) in petunia organs and tissues

Complementary DNA from poly(A) + RNA isolated from *P. hybrida* V26 anthers at developmental stages 2 and 3 (Fig. 1a) was synthesized with SuperScript III Reverse Transcriptase (Invitrogen) using an oligo(dT) primer. Degenerate primers UGT79B6G73f and UGT79B6F289r (Table S1) were based on the amino acid sequences, GAETT(A/S)D and ELT(D/G)LPF, respectively. PCR was performed using a *Taq* polymerase (Takara Bio Inc., Kusatsu, Japan) with thermal cycling conditions as follows: PCR mixture was incubated at 98 °C for 10 s, followed by 30 cycles of PCR (one cycle consists of 98 °C for 10 s, 42 °C for 30 s, and 72 °C for 1 min), and finally incubated at 72 °C for 3 min. The resultant product (ca. 700 bp) was cloned and sequenced.

### Quantitative reverse transcription PCR

RNA extraction and cDNA synthesis were performed as described previously (Yonekura-Sakakibara et al. 2007) using SuperScript™ III First-Strand Synthesis System (Invitrogen). Real-time PCR was performed as described previously (Yonekura-Sakakibara et al. 2014). The developmental stages of organs used for the analyses are shown in Fig. 1a. The primers, F3GalT9F_523F and F3GalT9R _587R for F3GalT, UGT79B31_196F and UGT79B31_257R for UGT79B31, Ph61074_1F and Ph61074_1R for Phcomp61074_c0_seq1, Ph27832_5F and Ph27832_5R for Phcomp27832_c0_seq1, and phSGN210759_1F and phSGN210759_1R for SGN210759 (Table S1) were designed using Primer Express software (Applied Biosystems). A dissociation program was used to confirm specific product formation. Plasmid DNAs containing the corresponding genes were used to create a calibration curve. The corresponding genes were amplified by PCR using primers, Phcomp61074_(−1)f and Phcomp61074_1390r for Phcomp61074_c0_seq1, Phcomp27832_(−1)f and Phcomp27832_1432r for Phcomp27832_c0_seq1, and PhSGN-U210759_131f and PhSGN-U210759_612r for SGN210759 (Table S1). Cloning of *UGT79B31* is described in the next section. Real-time PCR was performed on three biological samples.

### Cloning of UGT79B31 and in vitro assays

Full-length *UGT79B31* was obtained by PCR using KOD-Plus-Neo DNA polymerase (Toyobo Co. Ltd., Osaka, Japan), petunia pollen cDNA and primers Phcomp17948_(−1)f and Phcomp17948_1368r (Table S1); the amplification product was cloned into the pCR2.1-TOPO vector. The sequence of the resultant plasmid, pKYS479, was confirmed to exclude PCR errors. To construct the protein expression vector, full-length *UGT79B31* was further PCR amplified using primers, UGT79B31/pColdProS2f and UGT79B31/pColdProS2r (Table S1) and pKYS479 as a template; the amplification product was cloned into pColdProS2 using an In-Fusion Advantage PCR Cloning Kit (Clontech). After verifying the sequence of the resultant plasmid, pKYS491, the plasmid was transformed into *E. coli* strain BL21star™ (DE3). Production and purification of the recombinant protein were performed as described previously (Yonekura-Sakakibara et al. 2014). Transformed cells were grown at 37 °C until A600 reached 0.5. After addition of isopropyl-β-d-thiogalactopyranoside to a final concentration of 1 mM, cells were cultivated at 15 °C for 24 h. The cells were corrected and the protein was purified as a His fusion using TALON metal affinity resin (Clontech) according to the manufacturer’s instructions. The ProS2 tag was removed using HRV3C protease (Novagen) according to the manufacturer’s instructions. After exchanging the buffer for 50 mM Hepes–KOH, pH 7.5, proteins were concentrated using an Amicon Ultra filter (10,000 molecular weight cut-off; Millipore).

The standard enzyme assay reaction mixture was described previously (Yonekura-Sakakibara et al. 2012). The glycosyltransferase assay was performed at 30 °C according to Yonekura-Sakakibara et al. (2014). Flavonoid analyses were performed by UPLC/PDA/QTOF/MS as described above.

### Complementation of Arabidopsis *ugt79b6* mutants

The *UGT79B6* (At5g54010) promoter region was amplified with the primers At5g54010promoter-683 and At5g54010promoter-R (Table S1) and cloned into the pENTR/D-TOPO vector to construct the plasmid pKYS449 (Yonekura-Sakakibara et al. 2014). The coding region of UGT79B31 was amplified with the primers 79B6Pro-UGT79B31CDSf and UGT79B31CDS-r (Table S1) and fused to pKYS449 using an In-Fusion HD Cloning Kit (Clontech) to yield pKYS492 (pENTR/D-TOPO/683 bp fragments of the *UGT79B6* promoter fused to UGT79B31CDS). Plasmids pGWB1 and pKYS492 were used for LR reactions to construct the binary vector pKYS498 using Gateway LR Clonase™ II Enzyme Mix (Invitrogen). Plasmid pKYS498 (pGWB1/683 bp fragments of the *UGT79B6* promoter fused to UGT79B31 CDS) was used to transform *Agrobacterium* and Arabidopsis *ugt79b6* mutants as described previously (Yonekura-Sakakibara et al. 2014).

For selection of positive transformants, seeds were germinated on one-half-strength MS-agar medium containing 50 μg/ml kanamycin and grown for 10 days at 22 °C with a 16 h light/8 h dark photoperiod before positive transformants were moved to soil. Flowers from three individual F1 plants were harvested and analyzed by UPLC/PDA/QTOF/MS as described above.

### Subcellular localization

UGT79B31 was amplified with primers, GFP-79B31f/GFP-79B31r and 79B31-GFPf/79B31-GFPr (Table S1) for fusion to N- and C-terminal GFP, respectively; the amplification products were cloned into the pGWB5 or pGWB6 vectors (Nakagawa et al. 2007) using the Gateway Cloning System. The resultant plasmids pUGT79B31-GFP (UGT79B31 in pGWB5) and pGFP-UGT79B31 (UGT79B31 in pGWB6) were used to stably transform *Agrobacterium tumefaciens* strain GV3101 and transiently transform *N. benthamiana* leaves by the agroinfiltration method (Leuzinger et al. 2013). Plasmid pBIN61-P19 was used for the control (http://www.plantsci.cam.ac.uk/research/davidbaulcombe/methods/protocols/pbin61-p19.doc/view). Three days after infiltration, leaves were analyzed using a Zeiss LSM700 inverted confocal laser scanning microscope with a 40× dry objective (Zeiss). A diode laser with 488 nm excitation and appropriate filters (505–600 nm) were used to detect GFP fluorescence. ZEN 2011 software (Zeiss) was used for data analysis.

### Immunoblot analysis

Tobacco leaves (~ 10 mg) sampled 3 days after infiltration were ground in 150 µl sample buffer (Wako Pure Chemical Industries, Osaka, Japan) containing 5% β-mercaptoethanol. Samples were denatured at 98 °C for 2 min and proteins were separated by 10% polyacrylamide gel (SuperSep™Ace, Wako Pure Chemical Industries) electrophoresis. Proteins were transferred to PVDF membranes (Immobilon, Millipore) by semidry electroblotting. Blots were probed with anti-GFP primary antibody (SAB4301138, Sigma-Aldrich Co. LLC) and alkaline phosphatase conjugated anti-mouse secondary antibody (A3562, Sigma-Aldrich Co. LLC) and visualized using WesternBlue^®^ Stabilized substrate for alkaline phosphatase (Promega).

## Results

### In silico search of UGT(s) for pollen-specific flavonols

To identify the UGT(s) catalyzing the terminal glucosylation of pollen-specific flavonols, we conducted an in silico search of the Sol Genomics Network database (https://solgenomics.net/) using *UGT79B6* from *A. thaliana* as a query. To date, the identified flavonoid glycosyltransferases that catalyze glycosylation of the sugar moiety attached to flavonoid aglycones (GGTs) belong to the UGT79 and UGT94 families. The UGT79, UGT91, UGT92 and UGT94 families belong to the same orthologous group that contains genes derived from a common ancestor (Yonekura-Sakakibara and Hanada 2011; Yonekura-Sakakibara et al. 2014). Therefore, we focused on genes in the above UGT families as potential candidate genes. Four UGTs that belong to the UGT79, UGT91 or UGT94 subfamilies (Phcomp61074_c0_seq1, Phcomp17948_c0_seq2, Phcomp27832_c0_seq1, SGN-U210759) were identified as candidates. We also searched for UGT genes expressed in pollen by degenerate PCR using primers based on conserved regions [GAETT(A/S)D and ELT(D/G)LPF] among UGT79B6 and the homologs from *P. hybrida* (Phcomp61074_c0_seq1 and Phcomp17948_c0_seq2) (Table S1). The amplified product (668 bp) corresponded to Phcomp17948_c0_seq2. Phcomp61074_c0_seq1, Phcomp17948_c0_seq2 and Phcomp27832_c0_seq1 were designated as UGT79D1, UGT79B31 and UGT91S1, respectively, by the UGT nomenclature committee (Mackenzie et al. 1997; https://www.flinders.edu.au/medicine/sites/clinical-pharmacology/ugt-homepage.cfm). Thus, we obtained four candidate genes (*UGT79D1*, *UGT79B31*, *UGT91S1* and SGN-U210759) in total.

### Flavonol distribution in petunia organs/tissues and expression of candidate *UGT*s

**Fig. 2:**
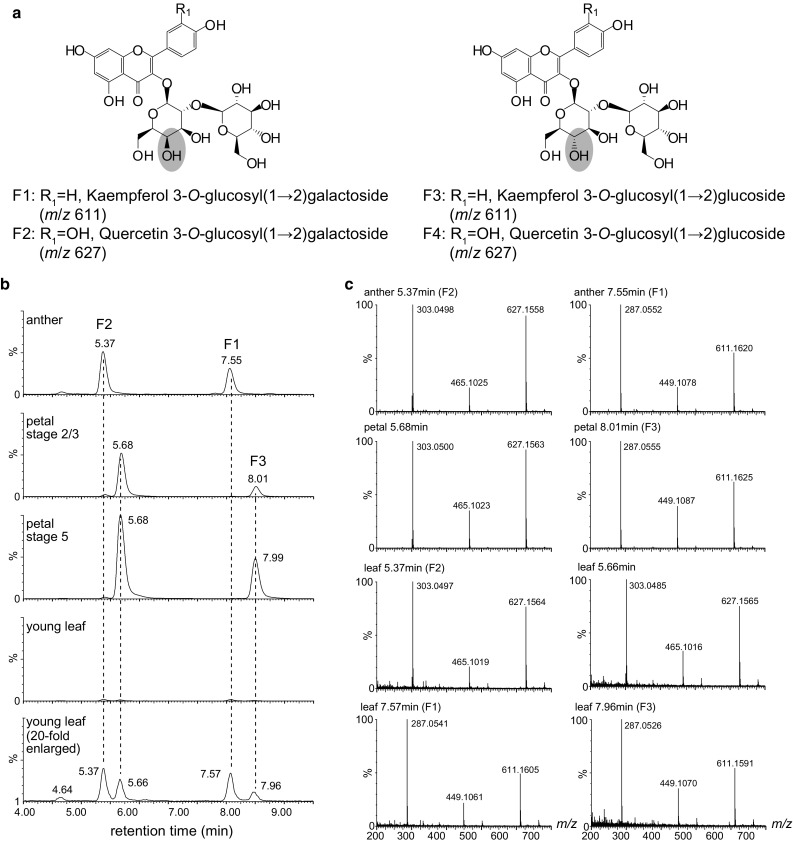
UPLC/PDA/QTOF/MS analyses of extracts from anthers, petals and leaves in *Petunia hybrida* V26. **a** Flavonol glycosides in petunia. **b** Extracted ion chromatogram at *m*/*z* 620 ± 10 was used to detect of flavonols. **c** Mass spectra of the major peaks are shown

Kaempferol/quercetin 3-*O*-glucosyl(1 → 2)galactosides accumulated exclusively in the pollen of *P. hybrida* V26 (Vogt and Taylor 1995). On the other hand, kaempferol/quercetin 3-*O*-glucosyl(1 → 2)glucoside derivatives were identified in petals and leaves of the *Petunia* cultivar ‘Mitchell’ (Bloor et al. 1998; Davies et al. 1998) and petals of *P.* × *hybrida* clone RL01 (Griesbach 1993). Flavonol 3-*O*-glucoside: 2″-*O*-glucosyltransferase/6″-*O*-glucosyltransferase also recognizes flavonoid 3-*O*-galactoside as a sugar acceptor at a comparable level (Yonekura-Sakakibara et al. 2014; Di et al. 2015; Rodas et al. 2016). These data suggest that the target gene encoding flavonol 3-*O*-galactoside: 2″-*O*-glucosyltransferase may be expressed in tissues other than pollen and may be involved in the 2″-*O*-glucosylation of flavonol 3-*O*-glucosides. To confirm the accumulation pattern of flavonol 3-*O*-glucosyl(1 → 2)galactoside/glucoside, the flavonoid distribution in anthers, petals and young leaves of *P. hybrida* V26 was determined. Kaempferol/quercetin 3-*O*-glucosyl(1 → 2)galactoside (F1/F2) were confirmed to accumulate in the anthers of *P*. *hybrida* V26 (Fig. 2). Kaempferol 3-*O*-glucosyl(1 → 2)glucoside (F3) and the compounds with an *m*/*z* value corresponding to quercetin 3-*O*-glucosyl(1 → 2)glucoside (F4) were detected in petals of *P. hybrida* V26, and a negligible amount of kaempferol/quercetin 3-*O*-glucosyl(1 → 2)galactosides (F1/F2), kaempferol 3-*O*-glucosyl(1 → 2)glucoside (F3) and the compound with the same *m*/*z* value corresponding to quercetin 3-*O*-glucosyl(1 → 2)glucoside (F4) were also detected in leaves (Fig. 2). These results suggests that the target UGT(s) may be expressed in petals in addition to pollen.

Expression profiles of the four UGT genes in various organs, tissues and developmental stages of petunia were analyzed by quantitative reverse transcription PCR (Fig. 1). The *UGT79B31* transcripts accumulated predominantly in petals and anthers. The expression profile of *UGT79B31* transcripts in anthers correlated well with the expression of pollen-specific *F3GalT*; however, expression of the other three *UGT* candidates did not correlate with *F3GalT* expression. *SGN*-*U210759* transcripts were accumulated exclusively in anthers at developmental stage 1. The transcripts of *UGT79D1* and *UGT91S1* were accumulated in anthers at a negligible level. The *UGT79B31* transcripts accumulated in petals and leaves in addition to anthers, consistent with the distribution patterns of flavonol 3-*O*-glucosyl(1 → 2)glycosides. Therefore, we selected *UGT79B31* for further analysis.

### In vitro characterization of recombinant UGT79B31

**Fig. 3:**
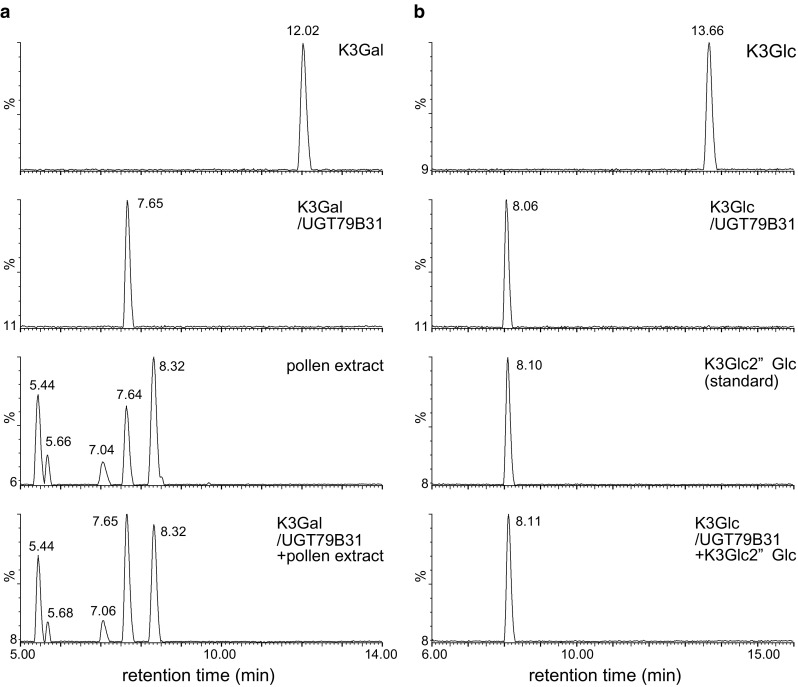
UPLC/PDA/QTOF/MS analyses of the reaction products of recombinant UGT79B31 protein. **a** Base peak chromatograms of kaempferol 3-*O*-galactoside (K3Gal), the reaction product of UGT79B31 with kaempferol 3-*O*-galactoside (K3Gal/UGT79B31), aqueous methanol extracts of *P. hybrida* pollen (pollen extracts) and K3Gal/UGT79B31 coeluted with pollen extracts (K3Gal/UGT79B31 + pollen extracts). **b** Base peak chromatograms of kaempferol 3-*O*-glucoside (K3Glc), the reaction product of UGT79B31 with kaempferol 3-*O*-glucoside (K3Glc/UGT79B31), kaempferol 3-*O*-glucosyl(1 → 2)glucoside (K3Glc2″Glc) and K3Glc/UGT79B31 coeluted with K3Glc2′′Glc (K3Glc/UGT79B31 + K3Glc2″Glc)

UGT79B31 was expressed as a His/ProS2-fusion protein in *E. coli* and was partially purified. After cleavage of the His/ProS2-tag, the UGT79B31 protein was used in enzyme assays. The recombinant UGT79B31 protein converted kaempferol 3-*O*-galactoside to kaempferol 3-*O*-glucosyl-(1 → 2)-galactoside as confirmed by comparable retention times and MS spectra as pollen kaempferol 3-*O*-glucosyl-(1 → 2)galactoside (Fig. 3, Fig. S1). Likewise, the glucosylation of kaempferol 3-*O*-glucoside to kaempferol 3-*O*-glucosyl-(1 → 2)-glucoside by UGT79B31 was confirmed (Fig. 3).

The specificity of UGT79B31 as a sugar acceptor was examined. UGT79B31 preferred flavonol 3-*O*-galactosides to the 3-*O*-glucosides. The activity with flavonol 3-*O*-rhamnosyl(1 → 6)glucosides were significantly lower than those for the 3-*O*-glucosides, suggesting that UGT79B31 had a low affinity for the 3-*O*-diglycosides as sugar acceptors (Table 1). The sugar donor specificity of UGT79B31 was also examined using kaempferol 3-*O*-galactoside or kaempferol 3-*O*-glucoside as potential sugar acceptors; in both cases, UGT79B31 highly preferred UDP-glucose to UDP-galactose. No UGT activity was detected for UDP-rhamnose, UDP-xylose, UDP-arabinose or UDP-glucuronic acid (Table 1). The effect of the end product, UDP, on enzyme activity was also investigated (Table 2). The enzyme was strongly inhibited by 1 mM UDP as with other flavonoid glycosyltransferases (Bar-Peled et al. 1991; Sawada et al. 2005). The UGT79B31 activity using kaempferol 3-*O*-galactoside or kaempferol 3-*O*-glucoside as sugar acceptors was inhibited by UDP in similar manner. Thus we identified UGT79B31 as a flavonol 3-*O*-glycoside: 2″-*O*-glucosyltransferase.

**Table 1:** Substrate specificity of recombinant UGT79B31 from *Petunia hybrida*

	Relative activity (%)
Sugar acceptor^a^	
Kaempferol (Kae)	ND
Kae 3-*O*-galactoside	100.0 ± 9.3
Kae 3-*O*-glucoside^b^	41.2 ± 4.2
Kae 3-*O*-rhamnosyl(1 → 6)glucoside	1.6 ± 0.5
Kae 3-*O*-glucoside-7-*O*-rhamnoside	31.5 ± 4.8
Kae 3-*O*-rhamnoside	Trace
Quercetin (Que)	ND
Que 3-*O*-galactoside	68.9 ± 15.7
Que 3-*O*-glucoside	45.6 ± 9.4
Que 3-*O*-rhamnosyl(1 → 6)glucoside	1.0 ± 0.8
Isorhamnetin 3-*O*-glucoside	31.0 ± 4.0
Cyanidin (Cya) 3-*O*-glucoside	10.0 ± 5.1
Cya 3-*O*-rhamnosyl(1 → 6)glucoside	ND
Cya 3-*O*-glucoside-5-*O*-glucoside	ND
Sugar donor^c^	
UDP-glucose	100.0 ± 4.6 (39.8 ± 2.7)^d^
UDP-galactose	1.4 ± 0.3 (1.2 ± 0.0)^d^
UDP-rhamnose	ND (ND)^d^
UDP-xylose	ND (ND)^d^
UDP-arabinose	ND (ND)^d^
UDP-glucuronic acid	ND (ND)^d^

The activity towards kaempferol 3-*O*-galactoside or UDP-glucose is taken to be 100%

*ND* not detected

^a^The reactions were performed with UDP-glucose as the sugar donor

^b^The enzymatic products were identified based on comparison with the standards. The specific activity of the recombinant UGT79B31 for kaempferol 3-*O*-glucoside and UDP-glucose is 941 ± 83.4 nmol min^−1^ mg^−1^ protein

^c^The reactions were performed with kaempferol 3-*O*-galactoside as the sugar acceptor

^d^The reactions were performed with kaempferol 3-*O*-glucoside as the sugar acceptor

**Table 2:** Effect of the end product, UDP, on UGT79B31 activity

	Concentration (mM)	Relative activity (%)
UDP	0	100.0 ± 2.6 (44.2 ± 0.5)
	0.001	94.3 ± 5.6 (42.3 ± 1.5)
	0.01	81.8 ± 0.4 (36.9 ± 0.5)
	0.1	27.8 ± 0.6 (17.6 ± 0.6)
	1	4.2 ± 0.1 (3.0 ± 0.2)

The reactions were performed with kaempferol 3-*O*-galactoside and UDP-glucose. The results using kaempferol 3-*O*-glucoside and UDP-glucose are shown in parentheses. Data are average values of three independent experiments with ± SD

### UGT79B31 functions as flavonol 3-*O*-glycoside: 2″-*O*-glucosyltransferase in planta

**Fig. 4:**
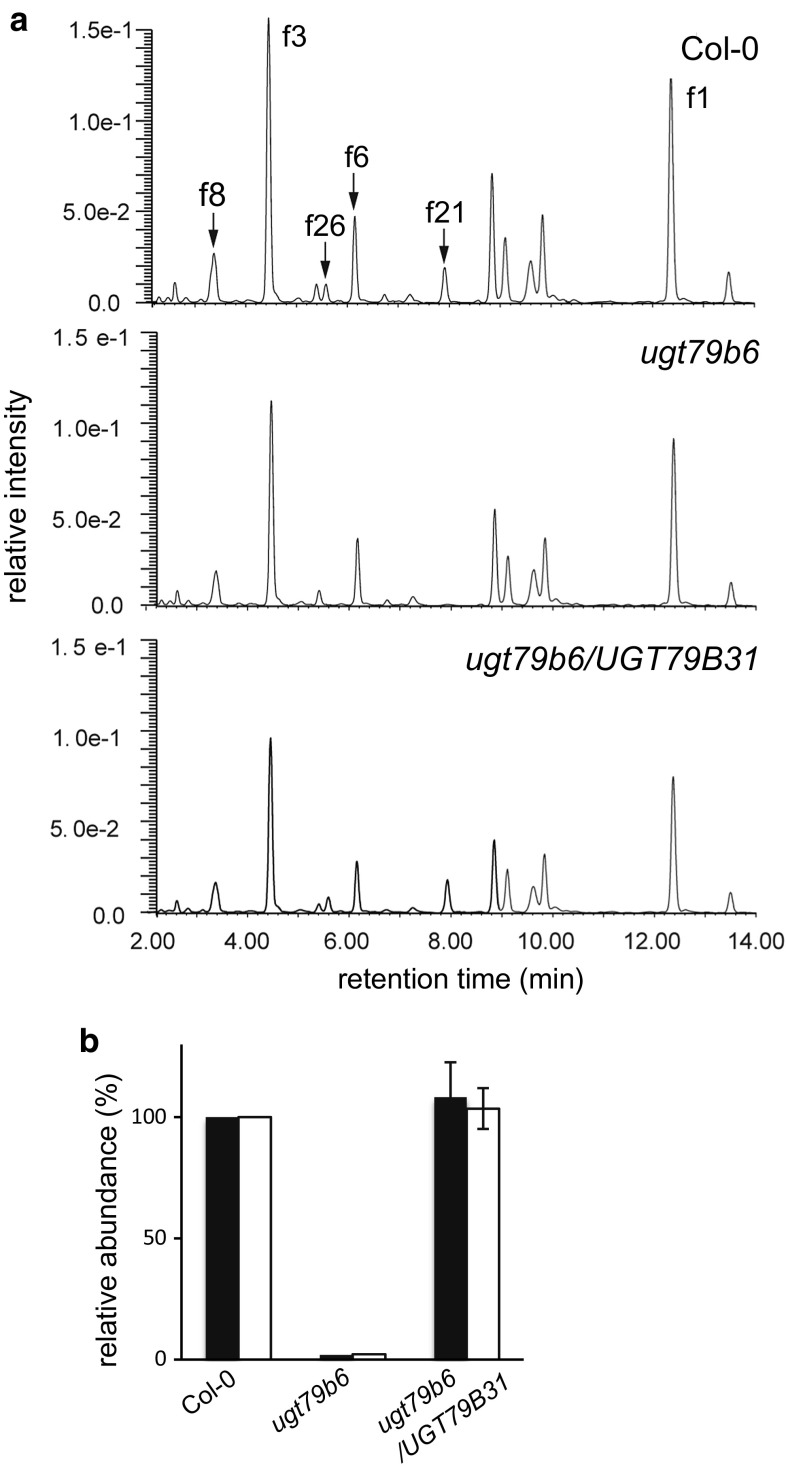
UPLC/PDA/QTOF/MS analysis of Arabidopsis wild type, the *ugt79b6* mutant and the *ugt79b6* transformed with *UGT79B31.* **a** Flavonol composition of flowers from wild type (Col-0), the *ugt79b6*-deficient mutant (*ugt79b6*) and the *ugt79b6* deficient mutant complemented with *UGT79B31* (*ugt79b6/UGT79B31*). f1 Kaempferol 3-*O*-rhamnoside-7-*O*-rhamnoside, f3 kaempferol 3-*O*-rhamnosyl(1 → 2)glucoside-7-*O*-rhamnoside, f6 quercetin 3-*O*-glucoside-7-*O*-rhamnoside, f8 quercetin 3-*O*-rhamnosyl(1 → 2)glucoside-7-*O*-rhamnoside, f21 kaempferol 3-*O*-glucosyl(1 → 2)glucoside, f26 quercetin 3-*O*-glucosyl(1 → 2)glucoside. **b** Relative abundances (%) of kaempferol 3-*O*-glucosyl(1 → 2)glucoside (f21, black bar) and quercetin 3-*O*-glucosyl(1 → 2)glucoside (f26, white bar) in wild type, the *ugt79b6* mutant and the complemented mutant line

To verify the function of UGT79B31 in planta, we used an Arabidopsis *ugt79b6* knockout mutant, *ugt79b6*-*3*, (Yonekura-Sakakibara et al. 2014) for functional complementation. UGT79B6 encodes flavonol 3-*O*-glucoside: 2″-*O*-glucosyltransferase and *ugt79b6* mutants lack pollen-specific flavonol 3-*O*-glucosyl-(1 → 2)-glucosides. UGT79B31 can utilize flavonol 3-*O*-glucosides in addition to the 3-*O*-galactosides as substrates in vitro, suggesting that UGT79B31 may complement the *ugt79b6* mutation. The *ugt79b6* mutants were transformed with the full-length *UGT79B31* cDNA coding region under the control of the Arabidopsis *UGT79B6* promoters (pKYS498). The flower flavonol profiles of transgenic *ugt79b6*-*3* lines transformed with pKYS498 were completely restored to that of wild type (Fig. 4). These data indicated that UGT79B31 functions as flavonol 3-*O*-glucoside: 2″-*O*-glucosyltransferase in planta.

### Phylogenetic analyses of UGT79B31 and petunia GGTs

**Fig. 5:**
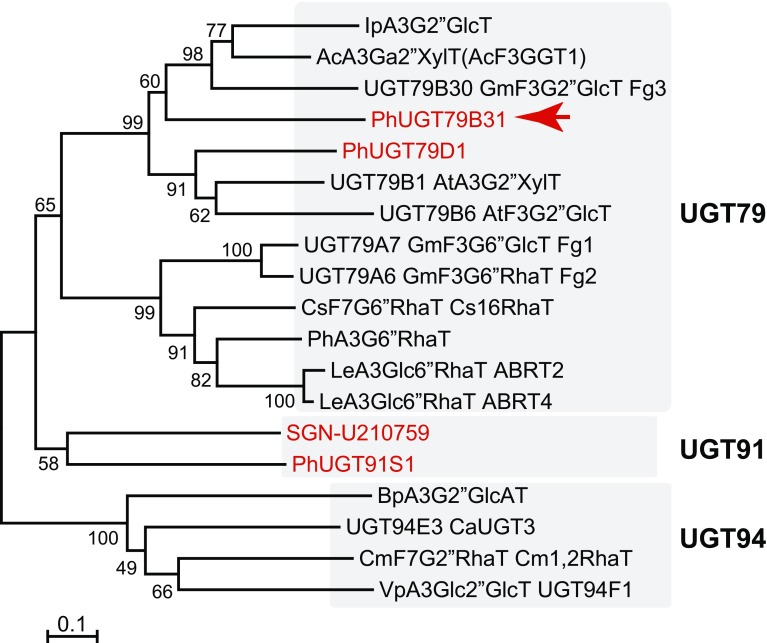
Neighbor-joining tree of known flavonoid GGTs and petunia candidate UGTs. The phylogenetic tree was generated using MEGA6 (version 6.06) (Tamura et al. 2013) as described previously (Yonekura-Sakakibara et al. 2014). The percentage of replicate trees in which the associated taxa clustered together in the boot strap test (1000 replicates) is shown next to the branches. The scale bar = 0.1 amino acid substitution per site. The Genbank accession numbers or AGI codes for the sequences are shown in parentheses: A3Glc6″RhaT ABRT2 (LC131336); A3Glc6″RhaT ABRT4 (LC131337); AcA3Ga2″XylT (FG404013); BpA3G2″GlcAT (AB190262); CmF7G2″RhaT Cm1,2RhaT (AY048882); CsF7G6″RhaT Cs1,6RhaT (DQ119035); IpA3G2″GlcT (AB192315); PhA3G6″RhaT (Z25802); UGT79B1 AtA3G2″XylT (At5g54060); UGT79B6 AtF3G2″GlcT (At5g54010); UGT79A6 GmF3G6″RhaT Fg2 (AB828193); UGT79A7 GmF3G6″GlcT Fg1 (LC126028); UGT94E3 CaUGT3 (AB443870); UGT79B30 GmF3G2″GlcT Fg3 (LC017844); VpA3Glc2″GlcT UGT94F1 (AB514127). *2″GlcAT* 2″-*O*-glucuronosyltransferase, *2″GlcT* 2″-*O*-glucosyltransferase, *2″RhaT* 2″-*O*-rhamnosyltransferase, *2″XylT* 2″-*O*-xylosyltransferase, *6″GlcT* 6″-*O*-glucosyltransferase, *6″RhaT* 6″-*O*-rhamnosyltransferase, *A3G* anthocyanidin 3-*O*-glucoside, *A3Ga* anthocyanidin 3-*O*-galactoside, *F3G* flavonoid 3-*O*-glucoside, *F7G* flavonol-7-*O*-glucoside. Abbreviations for species: *Ac Actinidia chinensis*, *At Arabidopsis thaliana*, *Bp Bellis perennis*, *Cm Citrus maximus, Ca Catharanthus roseus*, *Cs Citrus sinensis*, *Gm Glycine max*, *Ip Ipomoea purpurea*, *Le Lobelia erinus*, *Ph Petunia hybrida*, *Vp Veronica persica*

Generally flavonoid UGTs form a distinctive cluster based on the position of sugar acceptors they glycosylate. UGT79B31 belongs to the same cluster as other known flavonoid GGTs (OG8 cluster) and to a subcluster containing flavonoid glycoside: 2″-*O*-glycosyltransferases from the UGT79 family (Fig. 5). UGT79B31 had the highest amino acid sequence identity with anthocyanin 3-*O*-galactoside: 2″-*O*-xylosyltransferase, AcA3Ga2″XylT (55.7%) and slightly lower identity among other known flavonoid GGTs, IpA3G2″GlcT (52.5%), UGT79B30 (GmF3G2″GlcT, Fg3, 50.3%), UGT79B6 (F3G2″GlcT, 44.8%), UGT79B1 (A3G2″XylT, 44.6%), CsF7G6″RhaT (43.9%), UGT79A6 (GmF3G6″RhaT, Fg2, 41.5%), GmF3G6″GlcT (Fg1, 39.3%) and PhA3G6″RhaT (38.9%). The relatively lower sequence identity with flavonol 3-*O*-galactoside/glucoside: 2″-*O*-glucosyltransferase from soybean (UGT79B30, GmF3G2″GlcT, Fg3, 50.3%) suggests that flavonoid GGTs may gain their substrate specificity in a plant lineage-specific manner.

The commercial *P. hybrida* is derived from a white-flowered *P. axillaris* and a purple-flowered species of the *P. integrifolia* clade (Segatto et al. 2014). Recently, the genome sequences of two inbred laboratory accessions regarded as the parents of *P. hybrida*, *P. axillaris* N and *P. inflata* S6, were released (Bombarely et al. 2016). We conducted a BLAST search for petunia OG8 UGTs in the petunia genome databases (*P. axillaris* and *P. inflata,* Sol Genomics Network, https://solgenomics.net/) using UGT79B31, UGT92A1 and SGN210759 as queries. Most UGTs from *P. axillaris* and *P. inflata* are in a one-to-one correspondence, indicating that gene duplication of *UGT*s in OG8 did not occur frequently after the divergence of *P. axillaris* and *P. inflata* because the two species separated relatively recently. *UGT79B31* had the highest sequence identities with Peaxi162Scf00248g00003.1 (99.56%) and Peinf101Scf00058g07001.1 (98.02%) from *P. axillaris* and *P. inflata*, respectively, suggesting that these two genes may be *UGT79B31* orthologs.

### GFP fused to UGT79B31 localizes to the cytosol

**Fig. 6:**
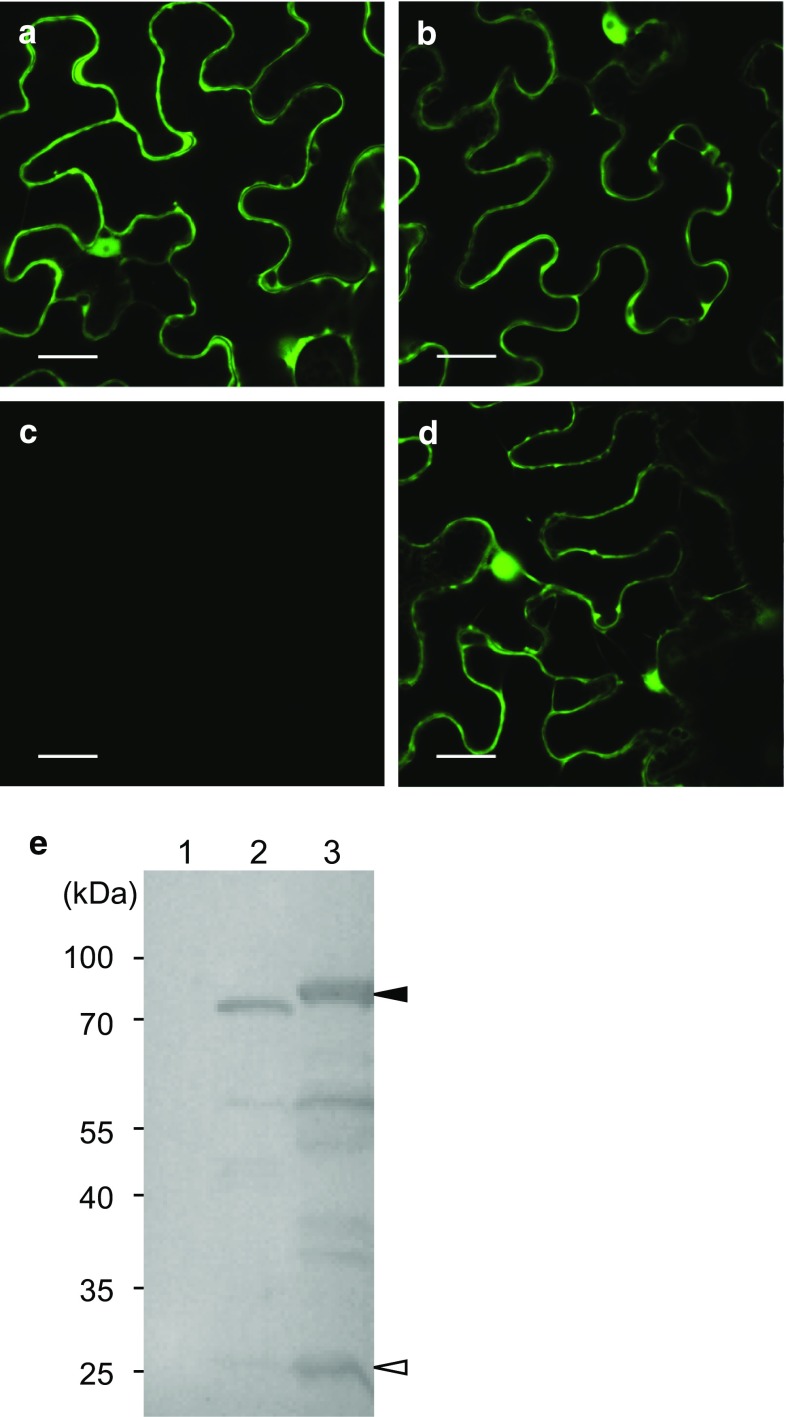
Confocal images of GFP fluorescence in leaves of *N. benthamiana* expressing UGT79B31-GFP (**a**), GFP-UGT79B31 (**b**), p19 control (**c**) and soluble GFP (**d**). Scale bar = 25 μm. **e** Immunoblot analysis of GFP fused to UGT79B31 protein in *N. benthamiana* transiently expressing the p19 control (lane 1), GFP-UGT79B31 (lane 2) or UGT79B31-GFP (lane 3). Fusion proteins were detected using anti-GFP antibody. The signals correspond to the molecular mass of UGT79B31-GFP (79.8 kDa) and GFP (26.9 kDa) are indicated with black and white arrowheads, respectively. The molecular masses (kDa) are given on the left

It has been suggested that F3GT, the enzyme corresponding to UGT79B31, may be a membrane-associated protein (Vogt and Taylor 1995). To investigate the subcellular localization of UGT79B31, GFP fused to the C- or N-termini of UGT79B31 (GFP-UGT79B31 and UGT79B31-GFP, respectively) were expressed transiently in *N. benthamiana* (Fig. 6). GFP fluorescence of GFP-UGT79B31 and UGT79B31-GFP was observed in the cytosol, as was the case with GFP only (Fig. 6). The protein detected by anti-GFP antibody corresponding to GFP-UGT79B31 (lane 2 in Fig. 6e) was slightly smaller than those corresponding to UGT79B31-GFP (lane 3 in Fig. 6e), suggesting that GFP-UGT79B31 may be truncated. GFP signals were also observed in the nuclei (Fig. 6a, b). The proteins smaller than 50–60 kDa were detected by anti-GFP antibody, suggesting that GFP signal in the nuclei may be caused by any cleavage products of GFP fused to UGT79B31. SignalP (Petersen et al. 2011) and TargetP (Emanuelsson et al. 2000) analyses reported that UGT79B31 has no chloroplast transit peptide, mitochondrial targeting peptide or secretory pathway signal peptide. The WoLF PSORT (Horton et al. 2007) analysis indicated that UGT79B31 localizes to the cytosol. These in silico analyses were consistent with our microscopic localization data.

## Discussion

### Flavonoid biosynthesis in pollen

Our results indicate that *UGT79B31* functions as a flavonol 3-*O*-glycoside: 2″-*O*-glucosyltransferase in planta and in vitro. The accumulation patterns of *UGT79B31* transcripts and flavonol glycosides in petunia tissues and organs suggest that kaempferol/quercetin 3-*O*-galactosides and the 3-*O*-glucosides are the predominant sugar acceptors for UGT79B31 in anthers and petals, respectively. These results also suggest that pollen-specific accumulation of kaempferol/quercetin 3-*O*-glucosyl(1 → 2)galactoside in petunia is first determined by a glycosylation step catalyzed by F3GalT, not UGT79B31. In contrast, UGT78D2 which catalyzes the first 3-*O*-glucosylation in Arabidopsis is distributed nearly throughout the plant and *UGT79B6* expression is specific to pollens (Yonekura-Sakakibara et al. 2014). Flavonol 3-*O*-diglycosides with a 1 → 2 inter-glycosidic linkage frequently accumulate as the major flavonoids in pollen of various plant species; however, the key enzymes determining the tissue/organ specificity of flavonol glycosides may be plant-species dependent.

Transient expression analyses using leaves of *N. benthamiana* showed that UGT79B31 fused to GFP localizes in the cytosol. Plant UGTs are thought to be localized in the cytosol (Bowles et al. 2006), and subcellular location prediction programs also support the cytosol localization of UGT79B31; however, petunia flavonol aglycones were proposed to be synthesized in the tapetum, released into the locule, and taken up in the cytosol of developing pollen grains to be glycosylated (Vogt and Taylor 1995; Taylor and Hepler 1997; Xu et al. 1997). F3GT has been postulated to be a membrane-associated protein based on its behavior in salt- or detergent-containing buffers (Vogt and Taylor 1995). In humans and yeast, two membrane-associated UGTs have been reported (Albesa-Jove et al. 2014). In addition, petunia F3GalT catalyzes the reverse reaction at a similar efficiency as the forward reaction (Miller et al. 1999). Further investigations, including tissue localization of F3GalT and UGT79B31 in developing anthers and mutants deficient in F3GalT and UGT79B31, are required to fully describe the pathways for flavonoid metabolism and the role of flavonoids in pollen.

#### *Author contribution statement*

KY-S conceived and designed research. EK participated in the experimental design. EK, SS, TM, RN and KY-S conducted the experiments and analyzed the data. EK, KS and KY-S wrote the paper. All authors read and approved the final manuscript.

## Electronic supplementary material

Below is the link to the electronic supplementary material.

Supplementary material 1 (EPS 1062 kb)

Supplementary material 2 (DOCX 25 kb)

## Abbreviations

F3GalT: Flavonol 3-*O*-galactosyltransferase
GFP: Green fluorescent protein
GGT: Flavonoid glycosyltransferase that glycosylates sugar moieties attached to flavonoid aglycones
UGT: UDP-glycosyltransferase
