# Supplementary material for: UGT79B31 is responsible for the final modification step of pollen-specific flavonoid biosynthesis in Petunia hybrida
Source: Planta. 2017 Dec 6;247(4):779–90. doi: 10.1007/s00425-017-2822-5 (PMC5856881; doi:10.1007/s00425-017-2822-5)
Supplement: Supplementary file 2 — Supplementary material 2 (DOCX 25 kb) [file 425_2017_2822_MOESM2_ESM.docx]

**Table S1** Primers used in this study

name sequences

*- degenerate PCR*

degenerateUGT79B6G73f 5′-GGIGCIGARACIACIKCIGA-3′

degenerateUGT79B6F289r 5′-AAIGGIARIYCIGTIARYTC-3′

*- Cloning*

Phcomp17948_(-1)f 5′-AATGGAGGACAAGAAGCTAACT-3′

Phcomp17948_1368r 5′- ATTACAATAGAGTTTGTAGCTTTTC-3′

Phcomp61074_(-1)f 5′- AATGGCAGAATCCATAGACTCA-3′

Phcomp61074_1390r 5′- TCTACTCAATTAGGAGACCATGC-3′

Phcomp27832_(-1)f 5’-TATGGAGATGGAGAATGACAAAG-3′

Phcomp27832_1432r 5′-CTCACTTAGAAAATTTGCATCC-3′

PhSGN-U210759_131f 5′- CATGACCACTAAGAACTACCATGT-3′

PhSGN-U210759_612r 5′- AGTACGTAAGGTTCCACCTTTAAG-3′

*-realtime PCR*

F3GalT9F_523F 5′-CGAATTACGGCTAGGTAGTTTGC-3′

F3GalT9R _587R 5′-CGGAAAATGGTGATTCCAAGTC-3′

UGT79B31_196F 5′- TGTTCGCGGTGAAGAATCAA-3′

UGT79B31_257R 5′- GAGGAAAACCAGGTGGAGGTT-3′

Ph61074_1F 5′- TCAAGTGCGGCTGAGCTAGTAC-3′

Ph61074_1R 5′- TGCAAAACCACAGCAGTTGAA-3′

Ph27832_5F 5′- GCTAGCCATGGGCCATATCA -3′

Ph27832_5R 5′- TGGCCTCTTTGAGTTAAGCACTT-3′

phSGN210759_1F 5′- TGTCCACTGCCTTCTTTAGTGTGT-3′

phSGN210759_1R 5′- CAACAGGTGGTCCGAGGTAAG-3′

*- protein expression*

UGT79B31/pColdProS2f 5′-AAGGTAGGCATATGGAGGACAAGAAGCTAACT-3′

UGT79B31/pColdProS2r 5′- GCTTGAATTCGGATCTTACAATAGAGTTTGTAGCT-3′

*- complementation*

At5g54010promoter-683 5′- CACCGGTCTAGTTGTTACTATTCG-3′

At5g54010promoter-R 5′- ATATGTTCTTCGGGGAAAATAAA-3′

79B6Pro-UGT79B31CDSf 5′-CCCCGAAGAACATATATGGAGGACAAGAAGC-3′

UGT79B31CDS- r 5′- GGCGCGCCACCCTTTTACAATAGAGTTTG-3′

*- tobacco infiltration*

GFP-79B31 f 5′-CACCGAGGACAAGAAGCTAACTTT-3′

GFP-79B31 r 5′-TTACAATAGAGTTTGTAGC-3′

79B31-GFP f 5′-ATGGAGGACAAGAAGCTAAC-3′

79B31-GFP r 5′-CAATAGAGTTTGTAGCTTTT-3′
